# Supplementary material for: Formation and Evaluation of a Two-Phase Polymer System in Human Plasma as a Method for Extracellular Nanovesicle Isolation
Source: Polymers (Basel). 2021 Jan 31;13(3):458. doi: 10.3390/polym13030458 (PMC7867002; doi:10.3390/polym13030458)
Supplement: Supplementary file 1 [file polymers-13-00458-s001.pdf]

Maria Slyusarenko et all., Formation and evaluation of a two-phase polymer system in human plasma as a method for extracellular nanovesicles isolation.

Supplementary Table S1. Assignment of peaks observed Raman spectrometry of pure components of plasma two-phase polymer system

Sample	Raman shift, cm^{-1}	Likely assignment	Ref.
UC-ENV	622	Phenylalanine (phenyl ring breathing)	[24]
	644	Tyrosine (C–C twisting)	[25]
	758	Tryptophan	[25,26]
	1004	Phenylalanine (phenyl ring breathing)	[24]
	1208	Phenylalanine, tyrosine (C-C6H5 stretching)	[27]
	1230, 1237-1241	Amide III bonds (rand coils and beta-sheet, respectively)	[27]
	1340	Tryptophan, lipids (CH2 deformation)	[28,29]
	1360	Tryptophan (Fermi resonance doublet with 1340 cm-1)	[28]
	1449	Lipids and proteins (CH2/CH3 deformation)	[30–32]
PEG	844, 860	CH2 rocking	[33,34]
	1141	CO stretch	
	1232	CH2 twist	
	1281	CH2 twist	
	1484	CH2 bending	
	546	C3C4, C4C5 deformation	[35]

Dextran	855	C-1-H bending	[36]
	922	COH deformation	
	1342	CCH deformation, OCH deformation, CO vibration	

24. Hernández, B.; Pflüger, F.; Kruglik, S. G.; Ghomi, M. Characteristic Raman Lines of Phenylalanine Analyzed by a Multiconformational Approach. *J. Raman Spectrosc.* **2013**, *44* (6), 827–833. https://doi.org/10.1002/jrs.4290.
25. Lee, C.; Carney, R. P.; Hazari, S.; Smith, Z. J.; Knudson, A.; Robertson, C. S.; Lam, K. S.; Wachsmann-Hogiu, S. 3D Plasmonic Nanobowl Platform for the Study of Exosomes in Solution. *Nanoscale* **2015**, *7* (20), 9290–9297. https://doi.org/10.1039/C5NR01333J.
26. Stone, N.; Kendall, C.; Smith, J.; Crow, P.; Barr, H. Raman Spectroscopy for Identification of Epithelial Cancers. *Faraday Discuss.* **2004**, *126*, 141. https://doi.org/10.1039/b304992b.
27. Notingher, I.; Verrier, S.; Haque, S.; Polak, J. M.; Hench, L. L. Spectroscopic Study of Human Lung Epithelial Cells (A549) in Culture: Living Cells versus Dead Cells. *Biopolymers* **2003**, *72* (4), 230–240. https://doi.org/10.1002/bip.10378.
28. Takeuchi, H. Raman Structural Markers of Tryptophan and Histidine Side Chains in Proteins. *Biopolymers* **2003**, *72* (5), 305–317. https://doi.org/10.1002/bip.10440.
29. Harada, I.; Miura, T.; Takeuchi, H. Origin of the Doublet at 1360 and 1340 Cm-1 in the Raman Spectra of Tryptophan and Related Compounds. *Spectrochim. Acta Part A Mol. Spectrosc.* **1986**. https://doi.org/10.1016/0584-8539(86)80193-3.
30. Enciso-Martinez, A.; Van Der Pol, E.; Hau, C. M.; Nieuwland, R.; Van Leeuwen, T. G.; Terstappen, L. W. M. M.; Otto, C. Label-free Identification and Chemical Characterisation of Single Extracellular Vesicles and Lipoproteins by Synchronous Rayleigh and Raman Scattering. *J. Extracell. Vesicles* **2020**, *9* (1), 1730134. https://doi.org/10.1080/20013078.2020.1730134.
31. Stremersch, S.; Marro, M.; Pinchasik, B.-E.; Baatsen, P.; Hendrix, A.; De Smedt, S. C.; Loza-Alvarez, P.; Skirtach, A. G.; Raemdonck, K.; Braeckmans, K. Identification of Individual Exosome-Like Vesicles by Surface Enhanced Raman Spectroscopy. *Small* **2016**, *12* (24), 3292–3301. https://doi.org/10.1002/smll.201600393.
32. Mohammadi, M. R.; Malkovskiy, A. V.; Jothimuthu, P.; Kim, K.-M.; Parekh, M.; Inayathullah, M.; Zhuge, Y.; Rajadas, J. PEG/Dextran Double Layer Influences Fe Ion Release and Colloidal Stability of Iron Oxide Nanoparticles. *Sci. Rep.* **2018**, *8* (1), 4286. https://doi.org/10.1038/s41598-018-22644-8.
33. Koenig, L.; Angood, A. C. Spectra of Poly(Ethy1ene Glycols) in Solution. *J. Polym. Sci. Part A-2* **1970**.
34. Zhbankov, R. .; Andrianov, V. .; Marchewka, M. . Fourier Transform IR and

Raman Spectroscopy and Structure of Carbohydrates. *J. Mol. Struct.* **1997**, *436–437*, 637–654. https://doi.org/10.1016/S0022-2860(97)00141-5.
35. Siew, D. C. W.; Cooney, R. P.; Taylor, M. J.; Wiggins, P. M. Vibrational Spectroscopic Studies of Aqueous Dextran Sulphate. *J. Raman Spectrosc.* **1994**, *25* (7–8), 727–733. https://doi.org/10.1002/jrs.1250250737.
